# Caffeine Consumption and General Health in Secondary School Children: A Cross-sectional and Longitudinal Analysis

**DOI:** 10.3389/fnut.2016.00052

**Published:** 2016-11-28

**Authors:** Gareth Richards, Andrew P. Smith

**Affiliations:** ^1^Autism Research Centre, Department of Psychiatry, University of Cambridge, Cambridge, UK; ^2^Centre for Occupational and Health Psychology, School of Psychology, Cardiff University, Cardiff, UK

**Keywords:** adolescent behavior, caffeine, energy drinks, health, mental health

## Abstract

Although caffeine is sometimes associated with beneficial effects in adults, the substance may be dangerous if intake is too high. This concern is particularly relevant in regards to children and adolescents, as consumption of energy drinks may be particularly high in such populations. For this reason, the current study examined data from the Cornish Academies Project to determine whether caffeine intake in secondary school children was related to responses to a single-item measure of general health. Two cross-sections of data were available: questionnaires were completed by 2030 at baseline, by 2307 at 6-month follow-up, and by 1660 at both time-points. Relationships were, therefore, explored both cross-sectionally and longitudinally. High caffeine consumption (i.e., 1000 mg/week) was associated with low general health in both cross-sections of data, and analyses of individual caffeine sources suggested that the effects related specifically to cola and energy drinks. However, after controlling for additional aspects of diet, demography, and lifestyle, total weekly intake only remained significantly associated with general health at the latter time-point. Further to this, null findings from cross-lag and change-score analyses suggest that caffeine and general health were unlikely to be causally linked in this sample. However, due to methodological limitations, such as the two cross-sections of data being collected only 6 months apart, it is suggested that further longitudinal and intervention studies are required in order for firm conclusions to be drawn.

## Introduction

Caffeine (1,3,7-trimelthyxanthine) is one of the most commonly consumed dietary ingredients (1), and the world’s most widely used psychoactive substance (2). Common sources are the kola nut (*Cola acuminate*), cacao bean (*Theobroma cacao*), guaraná berry (*Paullinia cupana*), and yerba maté (*Ilex paraguariensis*), though roasted coffee beans (*Coffea arabica* and *Coffea robusta*) and tea leaves (*Camelia siniensis*) are known to be the world’s primary sources of the substance (3). Through its action as an adenosine receptor antagonist, caffeine has a major effect on the central nervous system (4), and is also known to affect the cardiovascular system (5, 6), and diuresis (7).

Meta-analyses have found caffeine intake to be associated with increases in systolic and diastolic blood pressure [e.g., Ref. (8–10)]. However, when examining the effects in hypertensive individuals, Mesas et al. (9) found no support for associations between long-term coffee consumption and either increased blood pressure or increased risk of cardiovascular disease, although caffeine intake was shown to produce acute increases in blood pressure. Other meta-analyses have shown positive health effects of caffeine. Hernán et al. (11) for instance, found that coffee consumers were at reduced risk of Parkinson’s disease compared to non-consumers.

In addition to the above, a recent umbrella review of 91 meta-analyses (12) determined that coffee and caffeine intake are generally associated with health benefits in adults, although there were some exceptions to this rule, such as increased risks of low birth weight, pregnancy loss, and childhood leukemia. This review noted that coffee consumption was associated with probable decreases in risk of breast, colorectal, endometrial, and prostate cancers, cardiovascular disease, Parkinson’s disease, and type-2 diabetes. High probable decreased risk of liver cancer was also observed in men, and, although coffee was associated with increased risk of lung cancer, the effect was explained by smoking status. Probable no effects were observed in relation to risk of pancreatic and rectal cancer risk. Caffeine itself was associated with probable decreased risk of Parkinson’s disease, and type-2 diabetes. The discrepancies between findings relating to caffeine and those relating to coffee may be explainable by the presence of additional compounds found within caffeinated products. For instance, high levels of sugar may be associated with health problems, and are more likely to be found in energy drinks and cola than in tea and coffee.

Although caffeine appears, at least in some circumstances, to have positive effects on health, when too much is ingested intoxication can occur. Mild caffeine intoxications usually manifest as nausea and palpitations. In most cases, such adverse effects prevent further intake, reducing the risk of experiencing more serious complications. However, high doses of caffeine can cause a number of health concerns such as insomnia, gastroesophageal reflux, abdominal pain, hyperthermia, tachypnea, tachycardia, blood pressure lability, seizures, and even death (13, 14). Due to such concerns, it is useful to establish safe upper limits for consumption. The EFSA NDA Panel (15) stated that single doses of caffeine up to 200 mg, and habitual usage up to 400 mg/day do not give rise to safety concerns in adults. However, the report also concluded that there is currently insufficient information to determine safe intake levels for children and adolescents. It was, therefore, suggested that caffeine intakes of no concern derived from acute consumption in adults (i.e., 3 mg/kg bw per day) might provide a basis for determining such values in these populations.

Children and adolescents are known to commonly consume caffeine (16), and may also be particularly sensitive to its effects due to continued brain development, myelination, and synaptic pruning (17, 18). In addition, they are likely to be relatively naïve consumers, and may experience stronger effects due to lacking the level of tolerance typically found in adults. They may also consume similar portion sizes to adults, even though their bodyweight is on average considerably lower. Of particular concern therefore is the relatively recent introduction of energy drinks and shots to the consumer market. The reasons for such concern are that (i) caffeine levels in these products can be very high, (ii) chilled drinks are easier to consume quickly than hot drinks such as tea and coffee, and (iii) children make up a large proportion of consumers.

The research question that the current paper aims to address is whether caffeine intake is predictive of health problems in children and adolescents. More specifically, as young consumers may represent an at-risk group, it was hypothesized that high caffeine intake would be associated with low general health. It was also predicted that, should such a relationship be causal, the effect would be prevalent across time, and that increasing caffeine intake would be associated with decreasing general health. The current study proceeded with four basic aims: (i) to examine demographic and lifestyle correlates of caffeine intake in secondary school children, (ii) to determine whether caffeine intake is associated with self-reported general health, (iii) to ascertain whether caffeine intake is predictive of general health at 6-month follow-up, and (iv) to determine whether increasing caffeine intake over a 6-month time period is associated with decreasing general health.

## Materials and Methods

Data described in the current paper are from the Cornish Academies Project, which was a longitudinal study of a cohort of children and adolescents attending three secondary schools in the South West of England. Two cross-sections of data were collected: time 1 (T1) was collected 6 months prior to Time 2 (T2), and the research was conducted according to the guidelines laid down in the Declaration of Helsinki. The Cardiff University School of Psychology Ethics Committee approved all procedures (ethical clearance number: EC.12.09.11.3187), and written informed consent was obtained from each subject, as well as from their parents, prior to completion of the study.

### Participants

Three thousand seventy-one secondary school children were asked to participate in a two-part questionnaire survey, and 2610 agreed. Two thousand thirty completed the questionnaires at T1, and 2307 completed them at T2. One thousand six hundred sixty completed the survey at both time-points, giving an attrition rate of 18.23%.

### Demographic Data

Information relating to dietary intake, caffeine consumption, demographics, and stress, anxiety, and depression levels in this sample has already been discussed in previous publications (19–21). However, it is considered useful to provide a quick overview of the demographic background of the sample again here. Table 1, therefore, includes information relating to which academy and year group the children belonged, whether they were male or female, the presence/absence of special educational needs (SEN) status, eligibility/ineligibility to receive free school meals (FSM), ethnicity, whether or not English was spoken as an additional language, and whether or not the children were looked after by a non-parental guardian. Whereas previous publications have simply included the information relating to T1 or T2, Table 1 also includes descriptive statistics for those who only participated at T1, only participated at T2, participated at both time-points, or did not participate at either.

**Table 1:** **Frequency information for demographic variables at T1 and T2**.

		T1		T2		T1 only		T2 only		Both		Neither	
		*N*	%	*N*	%	*N*	%	*N*	%	*N*	%	*N*	%
Academy	1	954	31.1	971	29.2	109	29.5	51	7.9	574	34.6	229	52
	2	1363	44.4	1375	41.4	164	44.3	327	50.5	829	49.9	63	14.3
	3	754	24.6	977	29.4	97	26.2	269	41.6	257	15.5	148	33.6
Year	7	576	18.9	573	18.8	29	8.1	130	21.5	327	19.9	90	20.6
	8	601	19.8	602	19.7	66	18.5	142	23.5	327	19.9	66	15.1
	9	613	20.2	618	20.3	75	21	100	16.6	363	22.1	77	17.6
	10	613	20.2	616	20.2	98	27.5	118	19.5	300	18.2	97	22.2
	11	637	21	640	21	89	24.9	114	18.9	328	19.9	107	24.5
Sex	Male	1554	51.1	1018	48.5	179	50.1	274	47.3	822	50	250	57.2
	Female	1486	48.9	1079	51.5	178	49.9	305	52.7	823	50	187	42.8
SEN	Yes	669	21.8	899	29.2	85	23	190	30.5	308	18.6	156	35.7
	No	2399	78.2	2184	70.8	285	77	433	69.5	1352	81.4	281	64.3
FSM	Yes	396	13	398	13.1	59	16.5	67	11.1	186	11.3	85	19.5
	No	2644	87	2651	86.9	298	83.5	537	88.9	1459	88.7	352	80.5
Ethnicity	White	2938	97.3	2946	97.2	345	97.2	594	98.7	1592	97.5	411	94.3
	Not White	83	2.7	84	2.8	10	2.8	8	1.3	40	2.5	25	5.7
EAL	Yes	52	1.7	51	1.7	2	0.5	7	1.3	34	2	9	2.1
	No	3016	98.3	2868	98.3	368	99.5	545	98.7	1626	98	428	97.9
NPG	Yes	17	0.6	17	0.6	3	0.8	1	0.2	9	0.5	4	0.9
	No	3051	99.4	2909	99.4	367	99.2	563	99.8	1651	99.5	433	99.1

*“SEN” refers to special educational needs status, “FSM” refers to eligibility to receive free school meals, “EAL” refers to whether English is spoken as an additional language, and “NPG” refers to whether the child was looked after by a non-parental guardian. Data relating to participants who took part only at T1, at both time-points, or at neither time-point come from the first cross-section (i.e., T1); data relating to participants who took part only at T2 come from the second cross-section (i.e., T2)*.

The sex ratio was relatively balanced at both T1 and T2, and approximately 20% came from each year group of British secondary education (i.e., years 7–11). However, although similar numbers of children eligible to receive FSM were observed at both time-points, considerably more with a SEN status were present in the sample at T2 compared to T1. Most children who took part in the study were White, spoke English as their first language, and were not looked after by a non-parental guardian.

### Apparatus/Materials

The Diet and Behaviour Scale [DABS; (19)] was used to provide a measure of both the frequency and amount of consumption of common food and drink products that might have effects on psychological outcomes. This scale consists of 29 items; 18 record frequency of consumption on a five-point scale (1 = never, 2 = once a month, 3 = once or twice a week, 4 = most days [3–6], 5 = every day), 11 record the amount consumed per week or per day. Four of these latter items were used to calculate weekly caffeine intake from energy drinks, cola, coffee, and tea. Richards et al. (19) reported the DABS to be associated with a four-factor structure consisting of junk food, caffeinated soft drinks/gum, healthy foods, and hot caffeinated beverages. Subscale scores for the junk food and healthy foods factors are used as covariates in the current study; the other two factors were not controlled for due to their sharing variance with the predictor variable.

Three items were used to provide an indication of exercise participation; frequency of mildly energetic, moderately energetic, and vigorous exercise were all recorded on a four-point scale (1 = three times a week or more, 2 = once or twice a week, 3 = about once to three times a month, 4 = never/hardly ever). Finally, participants were asked to state how good they considered their general health to have been over the previous 6 months (1 = very good, 2 = good, 3 = fair, 4 = bad, 5 = very bad). This last question was chosen because it has been suggested that, when examined as an outcome variable, the best way to operationalize health status is by using a global single-item measure (22). Furthermore, single-item measures of self-reported health status have been used in population studies for over half a century, can reduce time-costs associated with multi-item measures, and have been shown to be significantly and independently predictive of a number of specific health problems, mortality, use of health services, changes in functional status, and recovery from ill health [see Ref. (23) for an overview].

### Design and Procedure

Teachers at three secondary schools administered pen and paper questionnaires to their students at both time-points in a classroom setting. Participants completed the questionnaires at the same time as those other students from their class who were taking part. Demographic information was then acquired directly from the School Information Management System (SIMS). These data, which were collected at both T1 and T2, included sex, age, school attended, school year, presence/absence of a SEN status, eligibility/ineligibility to receive FSM, ethnicity, whether English was spoken as an additional language, and whether or not the child was cared for by a non-parental guardian (do note however that sex and age were collected through SIMS at T1, but through questionnaire responses at T2).

### Statistical Methods

Total weekly caffeine intake was estimated from the number of cans of energy drink and cola, and cups of tea and coffee consumed per week. As the first aim of the current paper was to identify demographic and lifestyle correlates of caffeine consumption in adolescents, this variable was used to explore such relationships by using Spearman’s correlations, between-subjects *t*-tests and one-way analysis of variance (ANOVA).

The second aim of the current paper was to investigate associations between caffeine intake and general health. To do this, general health was dichotomized into high and low: those who answered with “very good” or “good” (answers 1 and 2) made up the high general health group, and those who answered with “fair,” “bad,” or “very bad” (answers 3, 4, and 5) comprised the low general health group. Total weekly caffeine intake was categorized into six groups: 0 mg/week, 0.1–250 mg/week, 250.1–500 mg/week, 500.1–750 mg/week, 750.1–1000 mg/week, and >1000 mg/week. This approach was identical to that used by Richards and Smith (21) when examining associations between caffeine intake and stress, anxiety, and depression in this same sample of secondary school children. The method was chosen as it allows for the detection of dose-dependent relationships and can also be used to establish a level of consumption above which an effect occurs, rather than simply demonstrating whether the variables are correlated. Previous studies (3, 24) have used intake above the 90th percentile to indicate heavy caffeine consumption. It is, therefore, worth noting that the highest intake group included in the current study (>1000 mg/week) represented a similar level of intake, with 90th percentile consumption being 1031 mg/week and 1054.3 mg/week at T1 and T2, respectively.

The above caffeine consumption groups were investigated cross-sectionally in relation to the dichotomous general health variable using Chi-square tests for linear association. Binary logistic regression (enter method) was then used to investigate these relationships further, while controlling for additional variance. Based on the methodology of Richards and Smith (21), the following covariates were entered into the models along with the predictor variables: diet (junk food and healthy foods DABS subscale scores; those for caffeinated soft drinks/gum and hot caffeinated beverages were not entered into the regression models due to them sharing considerable variance with caffeine consumption), demography (sex, school attended, school year, presence/absence of a SEN status, eligibility/ineligibility for FSM), lifestyle [average sleep hours, school attendance, exercise frequency; this latter variable was a factor analyzed score consisting of mild, moderate, and vigorous exercise, the details of which have been reported by Richards et al. (19)]. Note that although information relating to ethnicity, speaking English as an additional language, and being looked after by a non-parental guardian were recorded, they were not entered as covariates due to some of the groups being comprised of very small numbers of participants. Further to this, school year was included as a covariate rather than age. This was, first, to avoid multicollinearity (Spearman’s correlations determined that the two variables were very strongly correlated: T1 *r*[3013] = 0.972, *p* < 0.001; T2, *r*[2003] = 0.947, *p* < 0.001) and, second, because school year may capture additional variance, such as a child’s current Key Stage of secondary education. The analysis of caffeine and general health at T1 utilized covariates from T1, whereas the analysis from T2 used covariates from T2.

Health effects relating to caffeine could be attributable to trimethylpurinedione on the adenosine receptor or alternatively to other allelochemicals found in plants containing trimethylpurinedione. Therefore, it may be problematic simply to pool the effects of caffeine consumed from sources such as tea and coffee with those of cola and energy drinks. For this reason, it was deemed important to further investigate associations between general health and caffeine by separately examining each of the four sources of the substance measured by the DABS. Following the methodology used by Richards and Smith (21), participants were categorized as non-consumers, low consumers, or high consumers in the following manner: energy drinks (non, 0 mg/week; low, 0.1–133 mg/week; high, >133 mg/week), cola (non, 0 mg/week; low, 0.1–25 mg/week; high, >25 mg/week), coffee (non, 0 mg/week; low, 0.1–160 mg/week; high, >160 mg/week), and tea (non, 0 mg/week; low, 0.1–120 mg/week; high, >120 mg/week). Univariate level cross-sectional analyses were then conducted using Chi-square tests for linear association to help determine whether relationships between caffeine intake and general health might differ depending on the source from which the substance is obtained.

As two cross-sections of data were available, the third aim of the current paper was to investigate whether caffeine intake at T1 was predictive of general health outcomes 6 months later at T2. A Chi-square test for linear association was conducted, which was followed up with binary logistic regression. The same covariates (from T1) as used in the multivariate cross-sectional analyses were again included here.

A change-score analysis was conducted in order to address the fourth aim of the paper, to determine whether increasing caffeine consumption between the two time-points was associated with decreasing general health. This analysis proceeded with a Chi-square test at the univariate level, to examine whether increasing caffeine consumption (as opposed to not increasing caffeine consumption) was associated with decreasing (as opposed to not decreasing) in general health. This was then followed up with binary logistic regression, which again utilized the same covariates from T1 as used in the previous multivariate level analyses.

## Results

### Identification of Correlates of Caffeine Intake

As retrospective self-reporting of caffeine has been shown to be reliable [e.g., Ref. (25)], the DABS items that record the weekly number of cans of energy drink and cola, and cups of tea and coffee were used to calculate estimates of caffeine intake. The following values were assigned: can of energy drink (133 mg); can of cola (25 mg); cup of coffee (80 mg); and cup of tea (40 mg). The values for coffee, tea, and cola were chosen based on updated versions of those used by Brice and Smith (26), which were in turn based on those used by Barone and Roberts (3) and Scott et al. (27). The value for energy drinks was the mean caffeine content of the three products most commonly reported in the sample. The questionnaire did not consider differences in portion size, differentiate between instant and brewed coffee, or ask about the consumption of caffeine pills. The sum of caffeine intake from coffee, tea, energy drinks, and cola was calculated to provide an estimate of total weekly intake. The amount of caffeine consumed was found to be similar at both time-points: T1 *M* = 419.84 mg (SD = 526.76), T2 *M* = 421.77 (SD = 550). However, Ahluwalia and Herrick (16) have stated that studies of caffeine consumption typically report mean intake for either the whole sample or for all caffeine consumers, but not for both. It is, therefore, worth noting that the mean intake for those who used caffeinated products was markedly higher: T1 *N* = 1764, *M* = 471.25 (SD = 535.94), T2 *N* = 1973, *M* = 470.29 (SD = 560.79).

The current section aims to examine relationships between total weekly caffeine consumption and demographic and lifestyle variables. Significantly higher total weekly caffeine intake was observed in males compared with females, T1 *t*(1899.051) = 3.191, *p* = 0.001, T2 *t*(1948.766) = 4.262, *p* < 0.001, and in those who were eligible to receive FSM compared with those who were not, although this latter effect was only marginally significant at the first time-point: T1 *t*(1951) = 1.962, *p* = 0.05, T2, *t*(274.616) = 2.936, *p* = 0.004. Those with a SEN status were observed to consume significantly more caffeine than those without a SEN status at T2, *t*(2169) = 2.316, *p* = 0.021, although no such effect was detected at T1, *t*(1978) = 1.305, *p* = 0.192.

One-way between-subjects ANOVAs determined that caffeine consumption did not differ significantly between the three schools investigated, T1 *F*(2, 1979) = 2.044, *p* = 0.13, T2 *F*(2, 2199) = 0.741, *p* = 0.477, although Spearman’s correlations showed that intake was positively associated with school year, T1 *r*(1951) = 0.113, *p* < 0.001, T2 *r*(2146) = 0.221, *p* < 0.001. Caffeine intake was also negatively associated with average number of sleep hours, T1 *r*(1908) = −0.196, *p* < 0.001, T2, *r*(2115) = −0.199, *p* < 0.001, and school attendance, T1 *r*(1951) = −0.082, *p* < 0.001, T2 *r*(2150) = −0.157, *p* < 0.001, though no relationship was observed with exercise frequency, T1 *r*(1829) = 0.02, *p* = 0.381, T2, *r*(2055) = 0.016, *p* = 0.464.

### Associations between Caffeine Intake and General Health

Around 50% of the sample at both time-points reported their general health to be “good.” The majority of other respondents reported their health to be either “very good” or “fair,” and relatively few responded that their health was “bad” or “very bad” (for frequencies, see Table 2). For this reason, the measure was dichotomized into high and low, with those who answered “very good” or “good” (answers 1 and 2) making up the high general health group, and those who answered with “fair,” “bad,” or “very bad” (answers 3, 4, and 5) comprising the low general health group. This method allows for the risk of reporting less than ideal health to be assessed in relation to the amount of caffeine typically consumed.

**Table 2:** **Frequency of responses to the single-item measure of general health**.

	T1		T2	
	*N*	%	*N*	%
Very good	387	20.1	432	19.2
Good	1004	52.2	1157	51.4
Fair	447	23.2	547	24.3
Bad	68	3.5	101	4.5
Very bad	18	0.9	14	0.6

#### Cross-sectional Analysis

**Figure 1:**
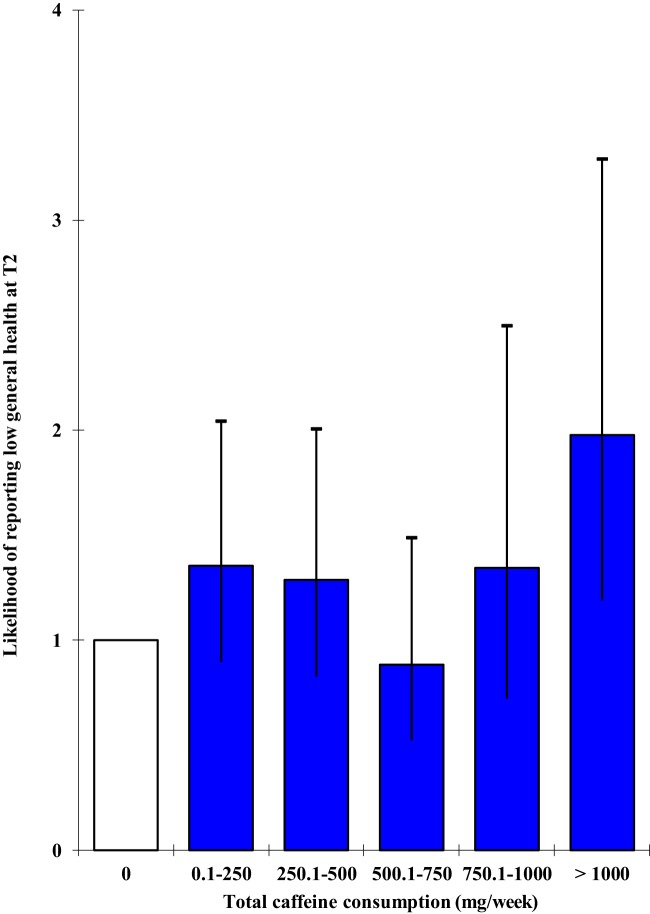
**Odds ratios and 95% confidence intervals for multivariate associations between total weekly caffeine intake and general health at T2**.

Significant negative relationships between total weekly caffeine intake and general health were observed at both time-points, which appeared mainly to reflect low general health being associated with consuming >1000 mg/week. However, at T2, high general health was also associated with non-consumption (i.e., the 0 mg/week group); for cross-tabulations, see Table 3. After controlling for covariates, binary logistic regression analysis determined that the overall association between total weekly caffeine intake and general health at T1 was no longer significant, Wald = 2.179, *p* = 0.824, and that none of the caffeine consumption groups differed significantly from the non-consumption group. However, the effect at T2 did remain significant, Wald = 12.848, *p* = 0.025, and reflected an increased risk of low general health being reported by the >1000 mg/week condition, OR = 1.978, 95% CI [1.188, 3.292], *p* = 0.009 (see Figure 1).

**Table 3:** **Univariate cross-sectional associations between total weekly caffeine intake and general health**.

			Total weekly caffeine intake					
			0 mg	0.1–250 mg	250.1–500 mg	500.1–750 mg	750.1–1000 mg	>1000 mg
General health T1	High	Count	155	543	302	147	86	130
		Expected count	148.3	540.4	292.3	149.8	89.7	142.5
		Column %	75.6	72.7	74.8	71	69.4	66
		Adjusted residual	1.1	0.3	1.2	−0.5	−0.8	−2.1
	
	Low	Count	50	204	102	60	38	67
		Expected count	56.7	206.6	111.7	57.2	34.3	54.5
		Column %	24.4	27.3	25.2	29	30.6	34
		Adjusted residual	−1.1	−0.3	−1.2	0.5	0.8	2.1
		χ2linear	5.021, *p* = 0.025							
	
General health T2	High	Count	175	614	332	179	87	144
		Expected count	159.1	620.6	325.2	171.8	87.3	166.9
		Column %	78.1	70.3	72.5	74	70.7	61.3
		Adjusted residual	2.5	−0.6	0.8	1.1	−0.1	−3.5
	
	Low	Count	49	260	126	63	36	91
		Expected count	64.9	253.4	132.8	70.2	35.7	68.1
		Column %	21.9	29.7	27.5	26	29.3	38.7
		Adjusted residual	−2.5	0.6	−0.8	−1.1	0.1	3.5
		χ2linear	8.043, *p* = 0.005							

When examining the effects of individual sources of caffeine, negative linear associations were observed between general health and caffeine from energy drinks and cola. In addition, high consumption of caffeine from tea at T2 was associated with above average general health, although the overall effect was not significant, and no such finding was made at T1. Caffeine intake from coffee was not associated with general health at either time-point. For cross-tabulations between caffeine intake from individual sources and general health at T1 and T2, see Table 4. When each of these caffeine sources were simultaneously entered into logistic regression analyses, few relationships of note emerged. High consumption of caffeine from cola was associated with low general health at both time-points, although the overall effect was only marginally significant at T1. In addition, a marginally significant association between low consumption of caffeine from tea and low general health was observed at T2. However, the overall effect was also only marginally significant, and was not detected at T1. For ORs, 95% CIs, and *p* values from these analyses, see Table 5.

**Table 4:** **Cross-tabulations between general health and weekly caffeine intake from energy drinks, cola, coffee, and tea**.

			Caffeine from energy drinks			Caffeine from cola			Caffeine from coffee			Caffeine from tea		
			0 mg	0.1–133 mg	>133 mg	0 mg	0.1–25 mg	>25 mg	0 mg	0.1–160 mg	>160 mg	0 mg	0.1–120 mg	>120 mg
General health T1	High	Count	826	306	249	486	466	418	981	190	212	579	396	408
		Expected count	810.2	296.6	274.2	457.9	459.3	452.8	975.3	190.9	216.9	586.2	379.9	416.9
		Row %	59.8	22.2	18	35.5	34	30.5	70.9	13.7	15.3	41.9	28.6	29.5
		Adjusted residual	1.6	1.2	−3.2	3.1	0.7	−3.8	0.6	−0.1	−0.7	−0.7	1.8	−1
	
	Low	Count	294	104	130	148	170	209	368	74	88	231	129	168
		Expected count	309.8	113.4	104.8	176.1	176.7	174.2	373.7	73.1	83.1	223.8	145.1	159.1
		Row %	55.7	19.7	24.6	28.1	32.3	39.7	69.4	14	16.6	43.8	24.4	31.8
		Adjusted residual	−1.6	−1.2	3.2	−3.1	−0.7	3.8	−0.6	0.1	0.7	0.7	−1.8	1
	
		χ2	10.545, *p* = 0.005			16.206, *p* < 0.001			0.533, *p* = 0.766			3.456, *p* = 0.178		
		χ2 linear	6.915, *p* = 0.009			15.653, *p* < 0.001			0.525, *p* = 0.469			0.01, *p* = 0.92		

General health T2	High	Count	949	345	269	561	556	448	1127	222	220	645	404	521
		Expected count	929.7	341.8	291.5	535.4	529	500.6	1120.9	214.9	233.2	648.9	420.6	500.5
		Row %	60.7	22.1	17.2	35.8	35.5	28.6	71.8	14.1	14	41.1	25.7	33.2
		Adjusted residual	1.8	0.4	−2.7	2.5	2.7	−5.3	0.6	1	−1.7	−0.4	−1.7	2.1
	
	Low	Count	365	138	143	194	190	258	459	82	110	273	191	187
		Expected count	384.3	141.2	120.5	219.6	217	205.4	465.1	89.1	96.8	269.1	174.4	207.5
		Row %	56.5	21.4	22.1	30.2	29.6	40.2	70.5	12.6	16.9	41.9	29.3	28.7
		Adjusted residual	−1.8	−0.4	2.7	−2.5	−2.7	5.3	−0.6	−1	1.7	0.4	1.7	−2.1
	
		χ2	7.417, *p* = 0.025			27.976, *p* < 0.001			3.482, *p* = 0.175			5.187, *p* = 0.075		
		χ2 linear	6.211, *p* = 0.013			20.326, *p* < 0.001			1.492, *p* = 0.222			1.795, *p* = 0.18		

**Table 5:** **Multivariate associations between individual sources of caffeine and general health**.

	Caffeine source	OR	95% CI	*p*-Values	
General health T1	Energy drinks	Low	0.843	0.615, 1.155	0.288
		High	1.206	0.873, 1.668	0.256
		Wald	3.582, *p* = 0.167		
	Cola	Low	1.093	0.802, 1.489	0.575
		High	1.419	1.029, 1.957	0.033
		Wald	5.168, *p* = 0.075		
	Coffee	Low	1.034	0.716, 1.493	0.859
		High	0.978	0.699, 1.368	0.898
		Wald	0.059, *p* = 0.971		
	Tea	Low	0.833	0.617, 1.124	0.232
		High	1.024	0.77, 1.363	0.869
		Wald	1.936, *p* = 0.38		
General health T2	Energy drinks	Low	1.01	0.754, 1.351	0.948
		High	1.038	0.745, 1.447	0.825
		Wald	0.049, *p* = 0.976		
	Cola	Low	0.897	0.673, 1.196	0.458
		High	1.434	1.071, 1.92	0.015
		Wald	11.557, *p* = 0.003		
	Coffee	Low	0.761	0.54, 1.073	0.119
		High	1.171	0.836, 1.641	0.357
		Wald	3.859, *p* = 0.145		
	Tea	Low	1.291	0.98, 1.701	0.069
		High	0.937	0.712, 1.234	0.643
		Wald	5.095, *p* = 0.078		

#### Cross-Lag Analysis

A Chi-square test for linear association did not uncover a significant association between total weekly caffeine intake at T1 and general health at T2, χ2 (1, 1584) = 0.216, *p* = 0.642. Likewise, no multivariate effect was observed through binary logistic regression analysis, Wald = 1.202, *p* = 0.945, and none of the caffeine consumption groups differed significantly from the non-consumption group. As pre-existing medical problems could potentially be a confounding factor, an additional logistic regression analysis was conducted in which general health at baseline (i.e., T1) was also entered as a covariate. As with the previous analysis, no association between caffeine intake at T1 and general health at T2 was observed, Wald = 1.8, *p* = 0.876, and none of the caffeine consumption groups differed significantly from the non-consumption group.

#### Change-Score Analysis

Percentage change scores for total weekly caffeine consumption and general health were calculated (using continuous variables) in the following manner: (T2 − T1)/T1 × 100. These variables were then recoded into three groups: “increase,” “decrease,” and “no change.” Nearly half (675, 43.9%) of participants reported no change in their general health, 445 (28.9%) reported that it had decreased, and 419 (27.2%) reported that it had increased. For caffeine intake, similar numbers of participants reported that their intake had increased (726, 46.9%) or decreased (682, 44.1%), and relatively few reported that it had not changed at all (140, 9%).

Caffeine consumption was then coded as, “increase” or “not increase” (the latter being comprised of those whose consumption had decreased or stayed the same between T1 and T2), whereas general health, was dichotomized as “decrease” or “no decrease,” with the latter group being comprised of those whose health had increased or stayed the same. The reason for coding the variables in this manner was that increasing caffeine consumption and decreasing general health were both considered to be undesirable, and so, Chi-square could be used to test whether the two were related. This test showed no significant association, χ2 (1, *N* = 1445) = 2.32, *p* = 0.128, and a subsequent binary logistic regression analysis confirmed the null finding, OR = 1.119, 95% CI [0.864, 1.448], *p* = 0.394.

## Discussion

The current paper identified a number of demographic and lifestyle correlates of caffeine consumption in British secondary school children. Further to this, caffeine usage was examined as a possible predictor of self-reported general health. Negative associations were initially observed in both cross-sections of data, though only one of these effects remained significant at the multivariate level. On closer inspection, the relationships appeared to relate specifically to caffeine consumed from cola and energy drinks. However, total weekly intake at baseline did not predict general health at 6-month follow-up, and change in caffeine consumption was not associated with change in general health. Doubt is, therefore, cast on the premise that the cross-sectional effects might be casual in nature.

### Demographic and Lifestyle Correlates of Caffeine Intake

Total weekly caffeine consumption varied considerably between participants, with mean intakes of 419.84 mg/week (SD = 526.76) and 421.77 mg/week (SD = 550) being observed at T1 and T2, respectively. Although these values are considerably lower than those reported in older populations [e.g., 1369.92 mg/week in US undergraduate students; (28)], caffeine consumption in a considerable proportion of participants is likely to have exceeded the recommendation of the EFSA NDA Panel (15) that children should avoid consuming >3 mg per kg of bodyweight per day. This in itself is concerning, as high consumption in young consumers has been associated with a range of undesirable outcomes, such as difficulty sleeping and morning tiredness (29), falling asleep at school (30), increased anger (31), violence and conduct disorder (32), and decreased school achievement (33).

The first aim of the current study was to identify demographic and lifestyle correlates of caffeine use in secondary school children. It was therefore interesting to note that caffeine consumption was higher in males than in females, and correlated negatively with average number of sleep hours. These findings reflect similar observations made in adult populations [e.g., Ref. (34, 35)]. In addition, children with a SEN status, and those eligible to receive FSM were also found to report elevated caffeine intake. A linear association was observed in which consumption increased throughout secondary school education, a trend that has previously been reported in children and adolescents (16). Caffeine intake was also negatively associated with school attendance, which may be a particular cause for concern considering the likely knock-on effects regarding academic attainment. Identifying such correlates in secondary school children is also useful because they can subsequently be controlled for when utilizing multivariate approaches to data analysis, thus reducing the chances of the true nature of effects being obscured by confounding factors.

### Associations between Caffeine Intake and General Health

The univariate cross-sectional analyses uncovered negative linear associations between total weekly caffeine intake and general health. These appeared to reflect increased occurrence of low general health in very high (>1000 mg/week) caffeine consumers, though an additional protective effect was observed in the 0 mg/week group at T2. However, although the association between caffeine intake and general health remained significant at T2 after additional dietary, demographic, and lifestyle covariates had been controlled for, the effect at T1 disappeared entirely. It is, therefore, not straightforward to interpret these findings. Further analysis of the individual sources of caffeine did, however, suggest that these negative associations relied on the consumption of caffeine from cola and energy drinks rather than from tea or coffee.

Although univariate associations between caffeine intake and general health existed cross-sectionally, total weekly consumption at T1 was not associated with general health at T2. In addition, no association was observed between changes in caffeine intake and changes in general health between the two time-points. These findings, therefore, suggest that the initial associations observed cross-sectionally are unlikely to have a causal basis. However, a possible explanation for these null findings is that, as mentioned above, caffeine and general health only remained significantly associated at the multivariate level in one cross-section of data. As the longitudinal analyses necessarily relied upon data from both cross-sections, this somewhat surprising finding might, therefore, have confounded the results.

The findings from the current study suggest that associations between caffeine intake and health in adolescents likely differ from those observed in adults. For instance, although an umbrella analysis conducted by Grosso et al. (12) reported that coffee and caffeine intake are typically associated with health benefits in adults, findings from the current study showed that high caffeine consumption was (at least at the cross-sectional level) associated with low general health, and that caffeine intake from coffee was not associated with health at all. The observation that caffeine consumed from cola and energy drinks appeared to explain the negative associations is also in line with the findings of a recent review article by Visram et al. (36). This paper showed that energy drink consumption in children and young people was associated with higher risk of health-damaging behaviors and physical health symptoms, such as headaches, stomachaches, insomnia, and hyperactivity, and suggested that such effects might be dose-dependent. Further research (both in relation to cola as well as energy drinks) is, therefore, required to better understand the nature of these effects.

### Limitations

Although an obvious advantage of the current research was that the samples examined were large, they were not entirely representative of the schools from which they came in regards to certain aspects of demography [representativeness of the samples has been discussed in previous publications; for T1, see Ref. (19); for T2, see Ref. (21)]. In addition, they related to a very specific population, both in terms of demography (e.g., age, socioeconomic background, ethnicity), and geographical location. For these reasons it is suggested that further research should aim to investigate the effects of caffeine on the health of adolescents in more representative samples. Further to this, adolescents commonly gain in size and weight during a 6-month period, and these variables were not controlled for in the current study.

An additional issue with the current study is that it relied upon a self-reported measure of general health. Although such measures can be reliable, they have rarely been used in samples of children, and even less so in relation to food/nutritional consumption. It is also possible that children might have misinterpreted the term “general health.” For instance, children might have considered small/medium injuries when responding to this measure, such as those picked up playing sports, which are unlikely to be related to diet. It is, therefore, questionable whether children and adolescents are able to answer such a question in a meaningful way.

The methodological nature of the study presented here meant that the longitudinal analyses faced a number of limitations not initially incurred at the cross-sectional level. One such problem is that only 6 months separated the collection of the two cross-sections of data. This close temporal proximity may have made it impossible to detect long-term effects of caffeine use over time. Future research could, therefore, address this issue by conducting longitudinal studies that leave a greater amount of time between initial data collection and subsequent follow-up.

Another limitation of the current study is that the samples at T1 and T2 differed more than was initially expected (which might also provide an explanation as to why the multivariate cross-sectional association between caffeine and general health was significant only at the latter time-point). In particular, the percentage of pupils with a SEN status was considerably higher at T2 (29.2%) compared with T1 (21.8%). This specifically reflected increases in the percentages of children with a SEN status present in Academies 2 and 3: Academy 2 T1 SEN = 17.1%, T2 SEN = 25.5%; Academy 3 T1 SEN = 26.1%, T2 SEN = 40.8% (the proportion of pupils with a SEN status at Academy 1 being 25.2% at both time-points). The large increase in percentage of participants with a SEN status from Academy 3 may also have reflected the observation that the number of pupils from this academy who were present in the sample increased by 223 between the two time-points; this was in stark contrast to Academies 1 and 2, which gained only 17 and 12 pupils, respectively. A likely explanation for this is that teachers at Academy 3 did not administer questionnaires to all classes at T1. In addition, some pupils may not have realized that they had consented to take part until the second time-point. This latter observation could therefore explain the relatively higher response rate at T2 (88.4%) compared with T1 (77.8%).

## Conclusion

The current study observed negative associations between caffeine intake and general health at the cross-sectional level, which appeared to be explainable by cola and energy drinks consumption. However, total weekly caffeine intake was not predictive of general health at 6-month follow-up, and changes in caffeine consumption were not associated with changes in general health. This would, therefore, suggest that the cross-sectional effects do not have a causal basis. However, due to the two cross-sections being collected only 6 months apart, and because the samples were shown to differ considerably, it is not possible to state with conviction that this is indeed the case. For this reason, further longitudinal studies that investigate the effects of caffeine over longer periods of time are required. Randomized controlled trials may also be posited as a more sophisticated method for determining whether caffeine consumption and general health are causally related in adolescents, or whether the variables merely happen to be correlated.

## Author Contributions

AS formulated the research question and designed and carried out the study; GR analyzed and interpreted the data and drafted the original manuscript. AS, then, revised the manuscript for important intellectual content, and both the authors approved the final version for publication. Both the authors agreed to be held accountable for all aspects of the work in ensuring that questions related to accuracy and integrity are appropriately investigated and resolved.

## Conflict of Interest Statement

The authors declare that the research was conducted in the absence of any commercial or financial relationships that could be construed as a potential conflict of interest.
